# MicroRNA expression profile of Lacrimal Glands in rabbit autoimmune dacryoadenitis model

**DOI:** 10.7150/ijms.50248

**Published:** 2020-10-16

**Authors:** Bo Liu, Lu Zhao, Yankai Wei, Sisi Chen, Lingzhai Bian, Di Guo, Min Gao, Hong Nian

**Affiliations:** 1 Department of Neurosurgery, Tianjin Medical University General Hospital, Tianjin, China.; 2 Tianjin Key Laboratory of Retinal Functions and Diseases; Tianjin International Joint Research and Development Centre of Ophthalmology and Vision Science; Eye Institute and School of Optometry, Tianjin Medical University Eye Hospital, Tianjin, China.

**Keywords:** Small RNA sequencing, autoimmune dacryoadenitis, Sjögren's syndrome dry eye, MicroRNAs

## Abstract

**Purpose:** To identify the differential expression of microRNAs (miRs) and the related gene networks and signal pathways in lacrimal glands (LGs) of rabbit autoimmune dacryoadenitis.

**Methods:** Autoimmune dacryoadenitis in rabbits was induced by transferring activated peripheral blood lymphocytes (PBLs). The LGs of normal and model group rabbits were collected for small RNA sequencing. The most differentially expressed miRs were validated by quantitative real time-polymerase chain reaction (qRT-PCR). Further, bioinformatics analysis including target gene prediction, Gene Ontology (GO) terms and Kyoto Encyclopedia of Genes and Genomes (KEGG) pathway enrichment analyses were performed.

**Results:** A total of 15 miRs were differentially expressed in the LGs of rabbit autoimmune dacryoadenitis relative to normal controls. GO and KEGG analysis revealed that most target genes of these dysregulated miRs were implicated in MAPK signaling pathway.

**Conclusion:** Our results showed for the first time the differentially expressed miRs and the related pathways involved in the pathogenesis of rabbit autoimmune dacryoadenitis. These results may contribute to elucidating molecular pathogenesis of Sjögren's syndrome (SS) dry eye.

## Introduction

Sjögren's syndrome (SS) dry eye is a chronic ocular autoimmune disease characterized by ocular inflammation and dysfunction in LGs, which may ultimately result in visual impairment 1, 2. SS dry eye is considered to be mediated by multiple factors including genetic, environmental and epigenetic factors 3. Although susceptibility to SS dry eye is in part genetic, and epigenetic modulation plays an important role in the development of various ocular conditions 4, 5, studies investigating the epigenetic factors that contribute to the SS dry eye are relatively scarce.

To explore the detailed mechanisms of SS dry eye, several animal models have been developed 6. Relative to other animal models, rabbit autoimmune dacryoadenitis, induced by injection of activated autologous PBLs, shares many common features with human SS 7. Moreover, the clinical features and LG histopathology of this autoadoptively transferred dacryoadenitis can persist for at least 6 months, indicating a chronic feature matching the chronic features of SS and often lacking in induced or genetically determined rodent dry eye models 8.

microRNAs (miRs), a class of small single-stranded, non-coding RNAs, are highly conserved key regulators of various autoimmune processes and can epigenetically regulate gene expression at post-transcription level through mRNA degradation or protein synthesis inhibition 9, 10. Recently, increasing evidences from clinical researches in patients and experimental animal models have shown that the dysregulation of miRs was closely related to the development of autoimmune diseases, including SS dry eye 11, 12. miRs have been shown to be differentially expressed in the tears, peripheral blood mononuclear cells (PBMCs) and salivary glands from SS patients 13-15. For example, Shi et al. demonstrated that the expression of miR-146a is upregulated and miR-155 is downregulated in PBMCs of patients with SS, and the expression levels of these miRs are correlated with the visual analog scale scores for dry eye 16. However, little is known about the miRs expression profile in LGs of SS dry eye 17.

In this study, we characterized the differential expression profile of miRs in LGs of rabbit autoimmune dacryoadenitis relative to normal ones and predicted their potential roles using bioinformatics methods. Our studies may provide clues to develop new clinical intervention strategies of SS dry eye.

## Materials and Methods

### Rabbit autoimmune dacryoadenitis model

Adult female New Zealand white rabbits (3.5-4 kg) were purchased from Vital River (Beijing, China) and were raised under pathogen-free conditions at Tianjin Medical University Eye hospital. The study was performed in accordance with the ARVO Statement for Use of Animals in Ophthalmic and Vision Research and approved by Institutional Animal Care and Use Committee of Tianjin Medical University.

All rabbits were subjected to a standardized ocular examination before the experimentation, and those with any pre-existing eye defects were excluded. Autoimmune dacryoadenitis was induced as introduced previously 18. Briefly, the left inferior LGs and peripheral blood were collected for isolating purified LG epithelial cells (pLGECs) and PBLs. After two days of culture, the irradiated pLGECs were co-cultured with autologous PBLs in a 1:1 ratio for activating PBLs. Five days later, PBLs were collected and adoptively transferred back into rabbits to induce autoimmune dacryoadenitis.

### Clinical and histopathological assessment

Clinical assessments including tear production, tear break-up time (BUT) and cornea fluorescein staining were performed every two weeks after injection of activated PBLs. Specific inspection method and scoring criteria were the same as those described previously 18. Rabbits were sacrificed at 6w and the right inferior LGs were dissected out for the histologic analysis. Tissues were fixated in 10% formalin, embedded in paraffin vax and sectioned. The sections were stained routinely with hematoxylin and eosin (H&E) to estimate inflammatory cell infiltration. The number of focus (aggregate of >50 lymphocytes) per 4 mm^2^ was recorded by two experienced technicians in a blinded fashion 18, 19.

### Small RNA-sequencing and data analysis

Total RNA of LGs in the normal and model group rabbits was extracted using the Trizol reagent (Invitrogen, Carlsbad, CA, USA) according to the manufacturer's instruction. The pooled RNA samples from the three rabbits in each group were subjected to Small RNA-sequencing and the Small RNA libraries were generated using the NEBNEXT library generation kit (New England Biolabs, Ltd., USA) following manufacturer's protocols in CapitalBio Corporation (Beijing, China). The library quality was assessed on an Agilent Bioanalyzer 2100 system and sequenced on the Illumina NextSeq Hiseq2500 platform (Illumina, San Diego, CA, USA).

For data analysis, clean reads were obtained by removing reads that containing adaptors and filtering low-quality reads. Then the clean reads were aligned to the reference genome by bowtie 20 and the sequences that matched pre-miRs were considered to be known miRs based on miRBase v22. After standardizing the value of reads by transcripts reads number per million (TPM) 21, the differentially expressed miRs between normal and model group rabbits were screened using the criteria of |FC| > 1.5 and false discovery rate (FDR) < 0.05.

### qRT-PCR

1 μg total RNAs were reverse transcribed using specific stem-loop primer. qRT-PCR was performed using FastStart Universal SYBR Green master (ROX) kit (Roche, Germany) on ABI 7900HT Real-Time PCR system (Thermo Fisher Scientific, Waltham, MA, USA). The amplification level was programmed with a denaturation step of 3 min at 95 °C, followed by 40 cycles of denaturation at 95 °C for 12s, and extension at 62 °C for 40s. For relative miRs expression level, all data were normalized to the expression level of U6. Data were calculated using the 2^[ΔCt(Control)-ΔCt(target)]^ method. Reverse transcription and qRT-PCR primers for miRs and U6 snRNA are listed in **Table 1.**

### miRs target gene enrichment analysis

The potential target genes of differentially expressed miRs were obtained from miRanda. Then a target genes network map was constructed using Cytoscape software v3.7.0. Functional annotation of potential target genes was performed by GO terms and KEGG pathway enrichment analysis with the threshold of corrected *p*-value <0.05.

### Statistical analysis

Data were analyzed with Graphpad prism 6.02 and presented as mean ± SD. The normality of the data was evaluated using Shapiro-Wilk test. When normality was not rejected, comparisons were made using Student's *t* test or two-way analysis of variance (ANOVA). *P*<0.05 is considered to be statistically significant.

## Results

### Clinical and histological examination of autoimmune dacryoadenitis

**Figure 1:**
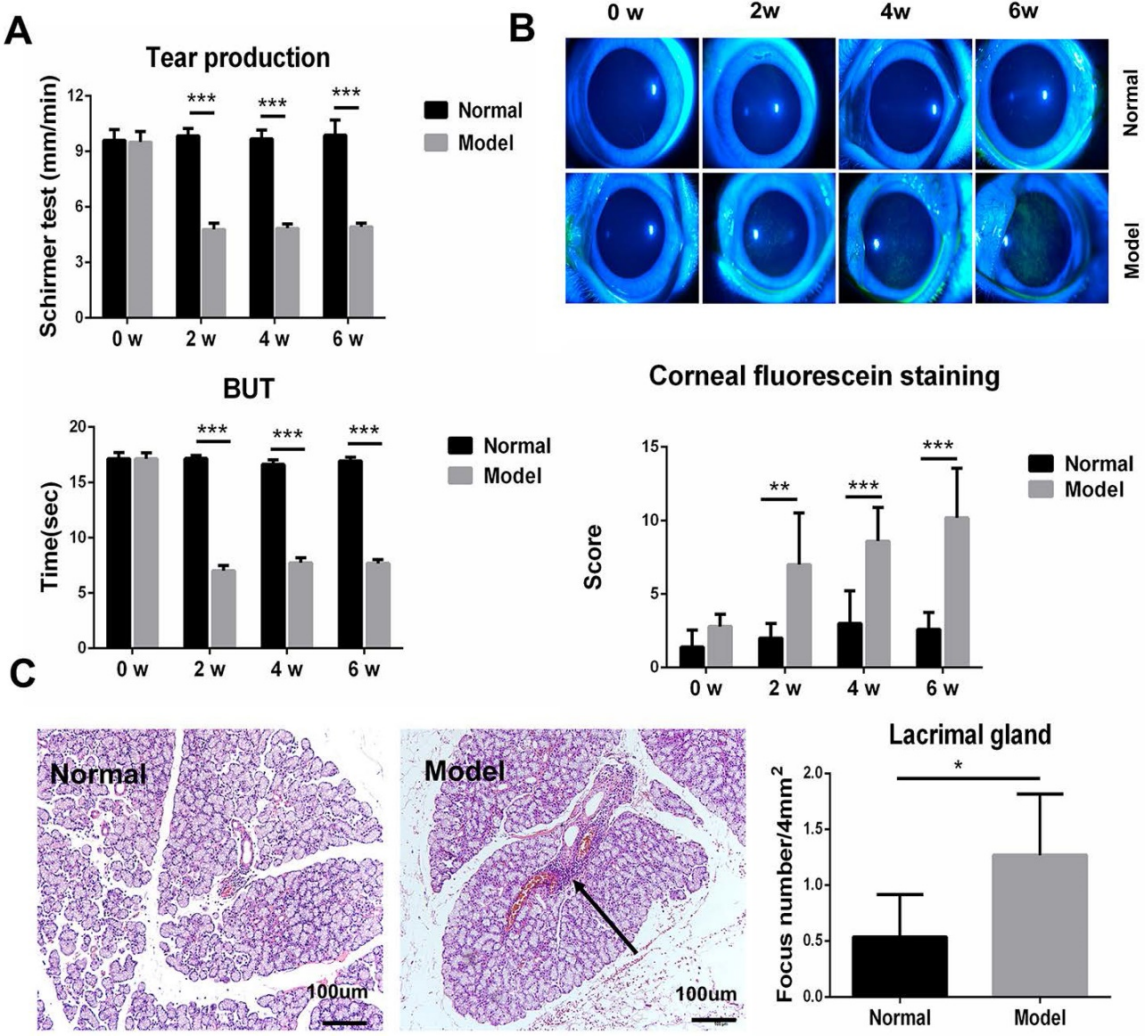
** Clinical ocular surface signs and histopathology analysis in rabbit autoimmune dacryoadenitis.** (**A**) The clinical scores of rabbits were assessed every 2 weeks in a blinded fashion after adoptive transfer of activated PBLs. (**B**) Representative photographs of fluorescein staining in model and normal rabbits. (**C**) Histologic analysis of LGs from normal and model group rabbits. Arrows shows perivascular or periductal lymphocytic foci. n=5 rabbits per group. Model group versus Normal group, *p<0.05, **p<0.01 or ***p<0.001.

Autoimmune dacryoadenitis was induced in rabbits by injection of activated autologous PBLs via ear margin vein. Clinical assessments were performed every 2 weeks and the histopathological examination was performed on week 6 after adoptive transfer (n=5/group). As shown in Figure 1A-B, the model group developed severe dry eye syndrome, characterized by decreased tear production, shortened tear BUT, and abnormal corneal epithelium staining, relative to the normal controls. Consistent with the clinical results, histological analysis of LGs revealed that inflammatory cell infiltration increased significantly in model group rabbits compared to the normal controls (Figure 1C).

### Identification of differential miRs profiles

**Figure 2:**
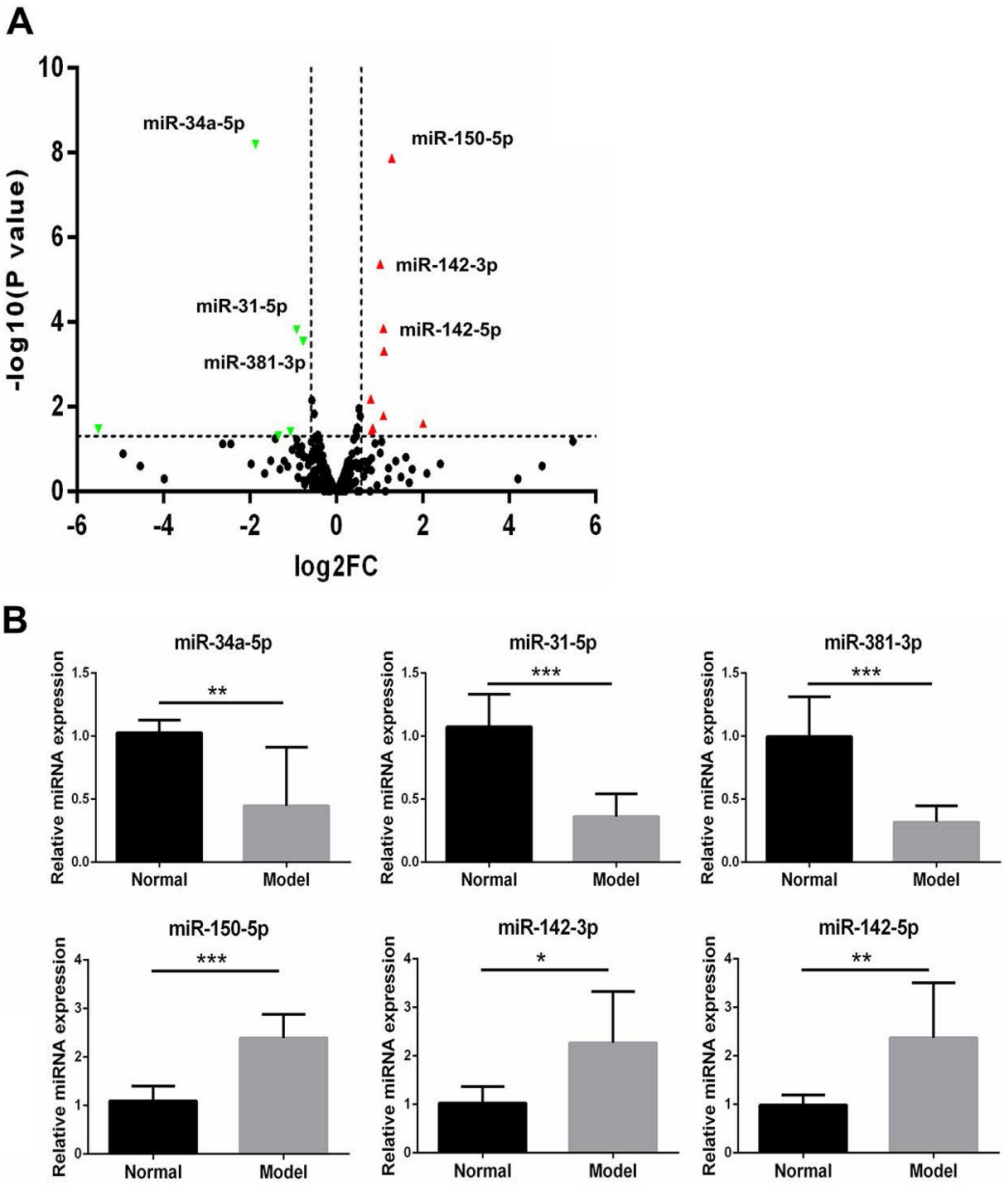
(**A**) The "volcano plot" picture of the differentially expressed miRs between normal and model group rabbits. Red triangles indicate upregulated miRs and green triangles represent downregulated miRs. (**B**) Validation of the expression patterns of the selected miRs using qRT-PCR. The relative expression of each miRs was normalized to U6 snRNA. The data shown are representative of at least three independent experiments and represented as mean ± SD. Statistically significant between normal and model group rabbits is indicated by *p<0.05, **p<0.01 or ***p<0.001.

To identify the difference of miRs expression in autoimmune dacryoadenitis, we employed small RNA-sequencing to analyze miRs profiles of LGs in model and normal rabbits. The results showed that the expression levels of 249 out of 367 miRs were modulated in LGs of autoimmune dacryoadenitis rabbits relative to normal ones. Using the threshold values log2FC>1.5 or log2FC<-1.5, and P<0.05, we identified 15 differentially expressed miRs including 9 upregulated (miR-150-5p, miR-142-3p, miR-142-5p, miR-335-3p, miR-144-3p, miR-188-5p, miR-671-5p, miR-378-5p, miR-653-5p) and 6 downregulated miRs (miR-34a-5p, miR-31-5p, miR-381-3p, miR-219a-5p, miR-656-3p, miR-432-5p) (Figure 2A).

### Validation of differentially expressed miRs with qRT-PCR

To confirm the small RNA-seq results, three upregulated miRs and three downregulated miRs among the filtered ones were validated with qRT-PCR between the model rabbits and normal samples (n=3/group). As shown in Figure 2B, the expression of miR-34a-5p, miR-381-3p and miR-31-5p was significantly decreased in model group rabbits compared with normal ones, whereas the miR-150-5p, miR-142-3p and miR-142-5p showed an up-regulated expression pattern, which was in agreement with the small RNA-seq results.

### Prediction of target mRNAs for differential expression miRs

**Figure 3:**
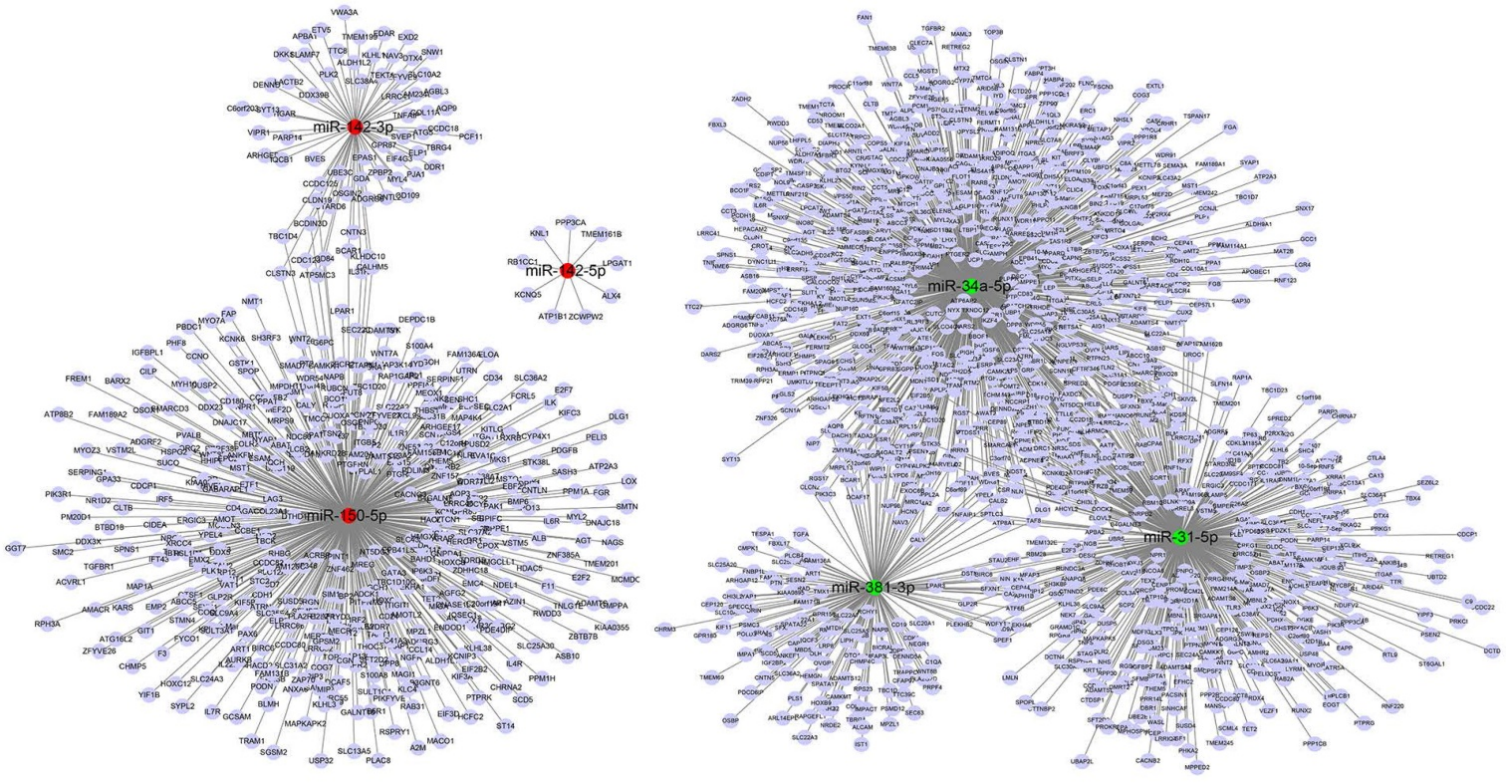
miRs-target genes networks of six most significantly differentially expressed miRs. Red indicates upregulated miRs, green indicates downregulated miRs, purple represents target genes.

The target genes of 15 differentially expressed miRs in rabbit autoimmune dacryoadenitis were predicted using miRanda database, and a total of 2215 target genes were obtained. As shown in Figure 3, 524 mRNAs were predicted as targets of miR-31-5p, 191 mRNAs were predicted as targets of miR-381-3p and 959 mRNAs were predicted as targets of miR-34a-5p. Moreover, both miR-31-5p and miR-381-3p target TNFAIP1, a protein that is derived from TNF and plays a key role in MAPK signaling pathway 22. Meanwhile, 513 genes were predicted as targets of miR-150-5p, in which MAPKAPK2 is activated by MAPK p38 and has been proved to be a therapeutic target for inhibition of chronic inflammatory diseases 23. Additionally, 77 genes were predicted as targets of miR-142-3p and 9 mRNAs were predicted as targets of miR-142-5p.

### GO enrichment analysis

**Figure 4:**
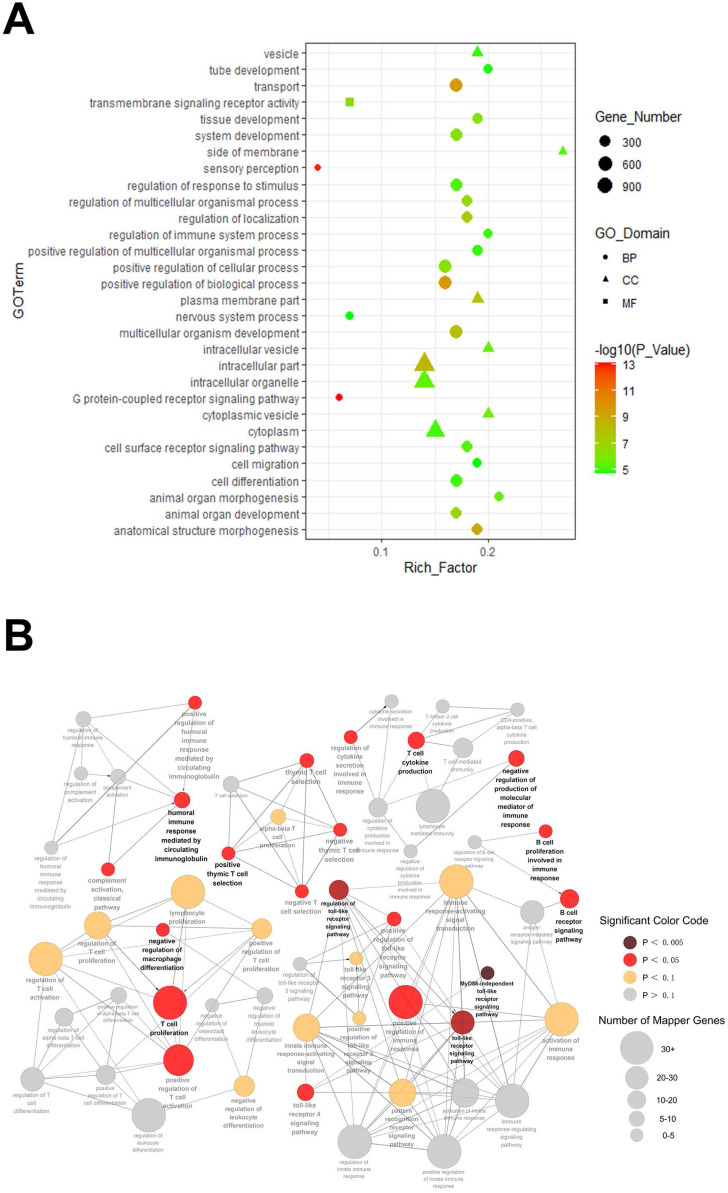
** GO terms enrichment analysis.** (**A**) GO analysis of predicted genes was performed in accordance with the BP, CC and MF. Shape represents GO categories, color corresponds to the -log10 of P value, size represents the enrichment gene number. (**B**) Predicted target genes were mapped for GO category: immune system process. Color represents the P value, and the diameter of each circle represents the number of genes enrichment.

GO enrichment analysis was performed to analyze the target gene functions. The top 30 GO terms in three categories are shown in Figure 4A. On the basis of biological process (BP), the top three processes were G protein-coupled receptor signaling pathway, sensory perception and transport. In molecular function (MF), target genes were significantly enriched in transmembrane signaling receptor activity. As for cellular component (CC), the top three processes were intracellular, intracellular part and plasma membrane part. To further understand the role of these altered miRs involved in the development of autoimmune dacryoadenitis, target genes were performed and mapped for GO category: immune system process. As shown in Figure 4B, the target genes of the differentially expressed miRs were significantly enriched in Toll-like receptor signaling pathway, regulation of immune response and T cell activation and proliferation.

### KEGG pathway analysis

**Figure 5:**
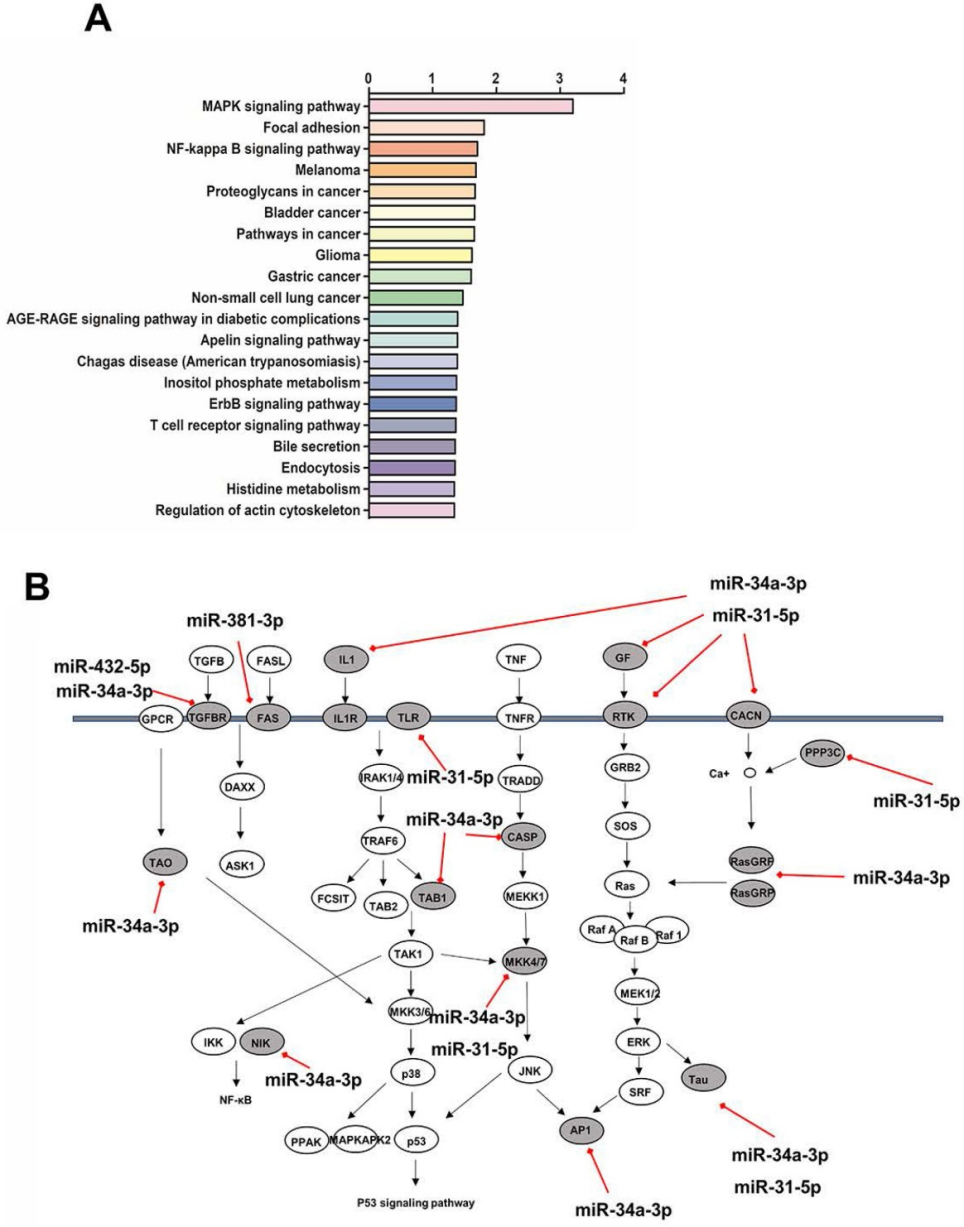
(**A**) Pathway analysis on target genes of differentially expressed miRs. The top 20 pathways are summarized. (**B**) miRs downregulated in autoimmune dacryoadenitis target multiple genes in the MAPK signaling pathway. The direct targets of downregulated miRs in the pathway are denoted with gray ovals.

KEGG pathway analysis showed the top twenty signaling pathways enriched with the target genes of the differentially expressed miRs (Figure 5A). The target genes in the model group rabbits were mainly enriched in MAPK signaling pathway, Focal adhesion, and NF-κB signaling pathway. We focused our attention on the MAPK signaling pathway, which is the most significant enrichment pathway and has been reported to be involved in the pathogenesis of SS dry eye. Remarkably, 18 genes at different level of MAPK signaling pathway were targeted by four identified downregulated miRs (Figure 5B).

## Discussion

SS dry eye is a multifactorial disease, including genetic predisposition, environmental and epigenetic factors 12. Recently, accumulating evidences have revealed that miRs are dysregulated in tears, PBMCs, and salivary gland of patients with SS 15, 17, 24. Nevertheless, the miRs profiling in LGs of SS dry eye remains unclear. In the present study, we performed small RNA-seq followed by qRT-PCR validation, and functional analysis to explore their role in autoimmune dacryoadenitis. Bioinformatics analysis revealed that some specific miRs might participate in the development of autoimmune dacryoadenitis via modulation of MAPK signaling pathway, Focal adhesion, and NF-κB signaling pathway.

In this study, we identified 15 differentially expressed miRs in LGs of autoimmune dacryoadenitis rabbits. Several of them have been reported to be involved in other inflammatory eye diseases. Guo et al. revealed that miR-150 and miR-653 were upregulated in peripheral blood lymphocytes in rats with experimental autoimmune uveitis 25. As for SS, previous studies reported that miR-335 was upregulated in the minor salivary gland of SS patients, and its expression level was inversely proportional to the unstimulated salivary flow rate 26. In addition, Johansson et al. identified miR-31 was reduced in the T cells of SS patients, which supports the autoimmune T cell response during chronic type I IFN exposure 27. These findings were consistent with our results that miR-335-3p was upregulated, whereas miR-31-5p was downregulated in autoimmune dacryoadenitis rabbits compared to the controls, suggesting these miRs may play a potential role in the pathogenesis of autoimmune dry eye. The identification of LGs specific miRs in our results may contribute to the pathogenesis of LGs dysfunction in SS dry eye.

To explore how these differentially expressed miRs participate in autoimmune dacryoadenitis pathogenesis, a miRs-mRNAs network was generated, which identified the potential link between miRs and their downstream targets. In this study, we note that the target genes of the significantly upregulated miRs, such as TGFβ2 and NFE2L2, may be closely associated with the pathogenesis of autoimmune dry eye. TGFβ2 is a crucial mediator of the ocular inflammation in dry eye disease 28. Besides, Stephen et al. found that the therapeutic effect of the cyclosporine emulsion on the chronic phase of autoimmune dry eye might be attributed to the increased expression of TGFβ2 29. NFE2L2 has previously been reported to affect cell migration and promote the corneal epithelial wound-healing process 30. Most recently, Matsuda et al. found RS9, a novel NFE2L2 activator, can induce the expression of NFE2L2-targeted genes, including NQO1 and GCLC, and ameliorate the symptoms of dry eye, implying the important role of NFE2L2 in the development of dry eye disease 31. With regards to the target gene of downregulated miRs, abnormal activation of P2RX7 has been revealed to participate in the development of autoimmune dry eye by inducing severe LGs dysfunction, and inhibition of P2RX7 may represent a promising therapeutic strategy for the management of autoimmune dry eye 32, 33. Therefore, we concluded that differentially expressed miRs may regulate the pathogenesis of autoimmune dacryoadenitis by modulating these target genes expression.

Our bioinformatics analysis showed that target genes of the differentially expressed miRs mainly enriched in MAPK signaling pathway. The MAPK pathways, including p38, extracellular signal regulated kinases (ERK) and c-jun N-terminal kinases (JNK), are pivotal regulators of various cellular activities, such as inflammation and innate immunity 34. Furthermore, MAPK signaling pathways have been reported to be critical for the production of inflammatory cytokines IL-1β and TNF-α, and matrix metalloproteinase (MMPs), which are involved in the pathogenesis of dry eye 34, 35. More importantly, Zoukhri et al. found the activation of JNK participated in IL-1β-induced impaired tear secretion in inflamed LGs 36. Besides, MAPK inhibitors such as SP600125 and SB203580 could ameliorate dry eye associated symptoms and reduce inflammatory cytokines production in tears of experimental dry eye disease, suggesting that inhibiting the activation of MAPK signaling pathway is beneficial to the treatment of dry eye 36, 37. Our results suggest that the downregulated miRs may participate in the pathogenesis of autoimmune dry eye by activating MAPK pathways.

The present study has several limitations. First, the number of samples was relatively small; and second, the target genes and signaling pathways of differentially expressed miRs were obtained from existing databases. These findings should be confirmed by further mechanistic studies. Additionally, the correlation between the degree of inflammation of autoimmune dry eye and miRs expression level could not be accurately investigated.

Taken together, our study identified changes in the expression of miRs in LGs of rabbit autoimmune dacryoadenitis and suggested the potential roles of these differentially expressed miRs in the pathogenesis of autoimmune dry eye. The present study here may provide important clues to the development of potential miRs-based therapeutic strategies for human autoimmune dry eye disease.

## Figures and Tables

**Table 1:** Primer sequences of stem-loop qRT-PCR

Name	Primer sequence(5'-3')		
	Reverse transcription	Forward	Reverse
ocu-miR-142-3p	GTCGTATCCAGTGCAGGGTCCGAGGTATTCGCACTGGATACGACTCCATA	CGCGCGTAGTGTTTCCTACTT	AGTGCAGGGTCCGAGGTATT
ocu-miR-142-5p	GTCGTATCCAGTGCAGGGTCCGAGGTATTCGCACTGGATACGACAGTAGT	GCGCGCATAAAGTAGAAAGC	AGTGCAGGGTCCGAGGTATT
ocu-miR-150-5p	GTCGTATCCAGTGCAGGGTCCGAGGTATTCGCACTGGATACGACCACTGG	GCGTCTCCCAACCCTCGTA	AGTGCAGGGTCCGAGGTATT
ocu-miR-381-3p	GTCGTATCCAGTGCAGGGTCCGAGGTATTCGCACTGGATACGACACAGAG	CGCGTATACAAGGGCAAGCT	AGTGCAGGGTCCGAGGTATT
ocu-miR-31-5p	GTCGTATCCAGTGCAGGGTCCGAGGTATTCGCACTGGATACGACACAGCT	CGAGGCAAGATGCTGGCAT	AGTGCAGGGTCCGAGGTATT
ocu-miR-34a-5p	GTCGTATCCAGTGCAGGGTCCGAGGTATTCGCACTGGATACGACACAACC	CGCGTGGCAGTGTCTTAGCT	AGTGCAGGGTCCGAGGTATT
U6	TTCACGAATTTGCGTGTCATC	CGCTTCGGCAGCACATATAC	TTCACGAATTTGCGTGTCATC

## Abbreviations

miRs: microRNAs
LGs: lacrimal glands
PBLs: peripheral blood lymphocytes
qRT-PCR: quantitative real time-polymerase chain reaction
GO: Gene Ontology
KEGG: Kyoto Encyclopedia of Genes and Genomes
SS: Sjögren's syndrome
PBMCs: peripheral blood mononuclear cells
pLGECs: purified LG epithelial cells
BUT: tear break-up time
MF: molecular function
CC: cellular component
BP: biological process
